# Improving big citizen science data: Moving beyond haphazard sampling

**DOI:** 10.1371/journal.pbio.3000357

**Published:** 2019-06-27

**Authors:** Corey T. Callaghan, Jodi J. L. Rowley, William K. Cornwell, Alistair G. B. Poore, Richard E. Major

**Affiliations:** 1 Centre for Ecosystem Science, School of Biological, Earth and Environmental Sciences, UNSW Sydney, Sydney, New South Wales, Australia; 2 Australian Museum Research Institute, Australian Museum, Sydney, New South Wales, Australia; 3 Ecology and Evolution Research Centre, School of Biological, Earth and Environmental Sciences, UNSW Sydney, Sydney, New South Wales, Australia

## Abstract

Citizen science is mainstream: millions of people contribute data to a growing array of citizen science projects annually, forming massive datasets that will drive research for years to come. Many citizen science projects implement a “leaderboard” framework, ranking the contributions based on number of records or species, encouraging further participation. But is every data point equally “valuable?” Citizen scientists collect data with distinct spatial and temporal biases, leading to unfortunate gaps and redundancies, which create statistical and informational problems for downstream analyses. Up to this point, the haphazard structure of the data has been seen as an unfortunate but unchangeable aspect of citizen science data. However, we argue here that this issue can actually be addressed: we provide a very simple, tractable framework that could be adapted by broadscale citizen science projects to allow citizen scientists to optimize the marginal value of their efforts, increasing the overall collective knowledge.

## Abstract 2

Citizen scientists collect data with distinct spatial and temporal biases, leading to unfortunate gaps and redundancies, and creating statistical and informational problems for downstream analyses. This Essay argues that by using a tractable framework which incentivizes looking, rather than finding, this issue can actually be addressed.

## Introduction

In October 2018, Corey traveled to Malaita, Solomon Islands, with the Australian Museum to conduct a biodiversity assessment with the local Kwaio people (https://australianmuseum.net.au/blog/amri-news/solomon-islands-ornithology/). While there, he submitted 66 eBird checklists, comprising 650 bird observations. He submitted the very first record of Malaita Dwarf-Kingfisher to the growing database—a database with >600 million observations comprising approximately 99% of the world’s bird species. Traveling to a remote part of the world to survey birds was truly a “once-in-a-lifetime” opportunity. But submitting eBird checklists from his smartphone—one example of a citizen science contribution—was simply part of his daily routine.

He is not alone. Citizen science is now mainstream, with hundreds of thousands of participants worldwide contributing observations of the natural world to various citizen science projects daily. Among the most popular projects, accumulating millions of observations annually, are those in which citizen scientists record the numbers and types of organisms observed [1,2]. But what is the difference between submitting an eBird checklist from a remote part of the world and submitting an eBird checklist while walking the dog near his home in Sydney, as Corey does most days? Is one inherently more “valuable” to the database than the other? In this paper, we examine this question, highlighting that not all citizen science observations are created equal. We argue that there is room for improvement in large-scale citizen science collection schemes and provide a conceptual framework to assign value to citizen science observations.

## Citizen science is mainstream

Citizen science projects—scientific research conducted in whole or in part by people for whom science is not their profession—are incredibly valuable for society [3], with their importance to scientific research growing each year [4]. These projects provide educational opportunities [5], increase scientific knowledge [6], and collect immense amounts of raw information about biodiversity in the world [7,8]. Citizen science data allow larger spatial and temporal scales for key research questions in many fields, including environmental toxicology, medicine, nutrition, astronomy, and biodiversity research [2,9]. In this essay, we focus on citizen science projects in which the main intent is to collect broadscale biodiversity data, but the arguments apply to any citizen science projects that sample in space and time.

Each citizen science project aimed at collecting broadscale biodiversity data falls along a continuum, from unstructured to structured, based on the objectives, survey design, flexibility, rigorousness, and detail collected about the observation process [10,11]. Projects with clear objectives, clearly planned data analysis, and rigorous protocols, for instance, are classified as structured projects. Conversely, projects with open and flexible recruitment and a general lack of protocols are classified as unstructured projects [10]. Many projects fall along this continuum and are thus classified as semistructured [10, 11]. Examples of such projects, and their associated level of structure, include iNaturalist (unstructured; [12]), eBird (semistructured; [7]), eButterfly (semistructured; [13]), FrogID (semistructured; [14]), and the UK Butterfly Monitoring Scheme (structured, [15]). Despite their level of structure in data collection, each of these projects has a specific aim: to collect observations of a unique taxon along with spatial and temporal data. We define one of these observations as a biodiversity sampling event (BSE).

A well-known feature of the data from these broadscale citizen science projects is the patchy distribution of BSEs across space and time [2,16,17], differing based on the level of structure of a project [10]. This leaves global citizen science datasets with spatial and temporal gaps and redundancies [18,19]. Other biases associated with citizen science projects include interobserver skill differences [20] and taxonomic biases [21,22], influencing the data validation/quality of a specific citizen science project [23]. Data collection biases can, in some cases, be minimized with certain statistical techniques [18,24–26]. For example, interobserver skill differences can be accounted for in species distribution models [24]. Or sampling strategies and protocols can be enhanced [18], whereby citizen science projects transform from unstructured to more structured projects throughout the life of the project [10]. Additionally, data can be filtered or subsampled to deal with error and uneven effort [27,28], pooled among species [29], and augmented with data with a known sampling effort [30]. More complicated machine learning and hierarchical clustering techniques also exist, allowing for investigation of the relative importance of a large number of explanatory variables [31–33]. Generally, sophisticated methods are preferred to simple methods when accounting for biases in citizen science data [25,34]. This is especially true for unstructured and semistructured citizen science projects, which collect some information on potential biases that can then be accounted for. Conversely, fewer biases need to be accounted for when using data from structured citizen science projects, and thus, simpler statistical techniques can be appropriate. But importantly, although some approaches can deal with biases for particular questions, none of these approaches can ultimately increase the information content in the data. This can only occur in the data collection process.

Different citizen science projects, based on the level of structure associated with data collection, necessitate different statistical approaches to minimize bias that arises from patchy biodiversity data. As an example, continental- and hemispheric-scale species distribution models derived using eBird data account for spatial bias by aggregating data in grids [35,36] while modeling differences in individual observer skill levels [20,24] of those who collect the data. These data are now being used to produce species-specific range maps with estimates of abundance (e.g., https://ebird.org/science/status-and-trends/). Data from the UK Butterfly Monitoring Scheme have produced reliable trend estimates for 62 butterfly species and accounted for sampling intensity by using a subsampling analysis [15]. There are a variety of methods to account for biases in citizen science projects at various parts of the data collection protocol. Biases have been minimized at the time of data collection by providing very structured protocols for projects that target specific monitoring areas and times—e.g., seagrass research [37]. Biodiversity data have been crowdsourced using an incentivized “reputation system” to motivate and reward participants who identify species, and critically, these data were ground-truthed by professionals, showing a 92% accuracy rate [38]. Hidden Markov models have been used to identify insect recordings in real time [39]. And many projects generally use active encouragement to collect large amounts of volunteered geographic information [40].

## Characterizing the value of biodiversity sampling events

Given the vast potential of citizen science monitoring schemes [2,41,42], methods to decrease patchiness and increase information in the data are crucial. Ultimately, this will help improve the confidence in downstream analyses. We foresee the following points to be critical in order to improve citizen science sampling for broadscale biodiversity projects:

- shift away from taxa-specific approaches and begin to incentivize looking in space and time, rather than finding
- implement a conceptual framework and associated algorithms that suggest high-marginal-value sampling sites to participants
- provide participants with incentive to contribute in the most meaningful manner

## Optimal sampling of biodiversity in space and time?

To maximize the value of each citizen scientist’s effort, we first have to answer a key question: Is a one-off trip to a remote part of the world more valuable than daily observations while walking a dog? Or, in general terms, what is the marginal value of each event to the overall project? This is specific to the questions researchers will ask using these data. Are the intended outcomes of the citizen science project aimed at producing reliable species distribution models? Or do the outcomes revolve around producing reliable population trends for a given management area? If the former, then a preference may be placed on homogeneous or stratified sampling in space, but if the latter, a preference might include less spatial sampling but longer time series at fewer sites. Inevitably, there are inherent trade-offs in spatial and temporal sampling, depending on the questions of interest. Projects with high spatial resolution of BSEs are beneficial for species distribution models [43,44], niche breadth [45], biodiversity measurements [9,46], and phylogeographical research [47]. Conversely, projects investigating detection probabilities [48], full-annual-cycle research [49,50], invasive species detection [51,52], and population trends [53,54] benefit from high-temporal-resolution BSEs.

Regardless of potential questions that will be asked by researchers, or intended goals of a citizen science project, there are some general principles in sampling design—relating to sampling in space and time—that can be applied to improve the structure of the data for many, if not all, future questions. We will first explore the relatively simplistic case of spatial resolution, followed by the more complicated instance of temporal resolution, before treating them both simultaneously.

### Spatial resolution

The simplest scenario for sampling global biodiversity would be to distribute BSEs homogeneously around the globe. Given species–area relationships [55,56] and the scale dependence of sampling biodiversity [57], the value of a BSE, given a preexisting set of BSEs, should be directly proportional to the distance between it and the nearest BSE. In other words, the information content that a given BSE adds to a collective dataset would be maximized by the distance between it and all other BSEs. But biodiversity is not homogeneous around the globe, and thus, BSEs should be stratified by habitat/biome, relative to overall biodiversity. Furthermore, organisms within and among taxa are not detected equally [58,59], making multiple BSEs at a given site (i.e., temporal replication) necessary for understanding local biodiversity [48,60], and habitat/biome definitions are debatable, suggesting that systematic sampling in space is neither achievable nor desirable.

### Temporal resolution

Temporal resolution, by necessity, may be thought of as analogous to an additional spatial dimension: temporal replication has to take place at a particular site. We do not provide a specific, rigid definition for site, as the definition will be highly dependent on the specific citizen science project. At the finest resolution, site could be equated to a particular BSE (i.e., unique latitude and longitude coordinates), or it could be a management unit of some spatial relevance (e.g., an urban greenspace, national park, county, state). If the latter, then spatial sampling would likely need to be applied within a specific “site” (i.e., multiple BSEs within a national park would be necessary). For better understanding of biodiversity changes, we should aim to increase the temporal replication of BSEs at a site. The sampling of every site can be visualized as a distribution that represents the sampling interval between BSEs. Wide variation will exist among sites, but the ultimate goal is to achieve a specific desired sampling interval between BSEs—left-shifting a particular site’s distribution of sampling intervals—or, in other words, decreasing the median and mean sampling interval for a site. Instead of many participants (or a single participant) visiting a single, well-sampled site (i.e., pseudoreplication), the visitation of a site can be optimized so that the tail of the distribution of all sites is left-shifted. Thus, the value of a BSE at a site would be related to the desired sampling interval and the time since the last sample. In other words, a BSE at a site that hasn’t been sampled in a month is marginally more valuable than a BSE at a site that was sampled the previous day, dependent on the desired sampling interval at a site. Marginal values are dynamic, as new BSEs are continuously submitted to a citizen science project.

### Spatial and temporal resolution

The ultimate goal in the future of broadscale biodiversity citizen science projects should be to increase spatial resolution while simultaneously increasing temporal replication at sites, balancing inherent trade-offs in spatial and temporal resolution. Ultimately, ecological and conservation outcomes that combine both spatial and temporal data can be achieved [9,61]. And information at spatial and temporal scales are the necessary types of data for broadscale conservation prioritization.

## Forecasting the value of future BSEs

Here, we provide a simple but general framework to forecast the marginal value of future BSEs. This framework requires a desired outcome: defining which specific information about a species or group of species is important for conservation or basic science (e.g., species distribution models or trend detection). There could be many desired questions, dependent on specific management goals. The management goals can also define the species or species pools of special interest—for example, all migratory birds in a national park or one specific species that is highly threatened. That goal defines a statistical model, and then, within that model, the contribution of each individual BSE can be quantified using the statistical concept of leverage [62]. High-leverage BSEs are useful for the desired outcome in that they are very important (i.e., influential) observations for the model, whereas low-leverage BSEs are less useful. The goal of future sampling can then be defined precisely: encourage a shift from low-leverage, low-value BSEs to high-leverage, high-value BSEs.

Of course, the distribution of biodiversity in time and space is not deterministic, and so we cannot predict the exact leverage of future BSEs. We can, however, predict the “expected leverage.” To find this, we look at the past: because the desired outcome is an improved statistical model, then for past data, it is simple to calculate the leverage for each BSE. To determine the effect of space and time on statistical leverage in the past, leverage values can be regressed against a suite of potential spatial and temporal variables, which are likely to influence the outcomes of the intended statistical model. Examples of forward-looking parameters we find important in space and time include (1) whether the site was sampled, (2) the distance to the nearest sampled site, (3) the median sampling interval of BSEs, (4) the median sampling interval of the site’s nearest neighbor, and (5) days since the last BSE at the site. A number of other variables could also be included in this framework, including observer skill, time of day, and weather, but we focus on the variables generalized across varied structured to unstructured citizen science projects.

In S1 Text, we present a stepwise approach to calculating these variables and, thus, the marginal value of a given site in space and time. The actual parametrization of these variables will depend on the statistical model of interest. For example, for a species distribution model, the highest-valued BSE is likely to be the furthest away from other BSEs, whereas for phenological questions, the highest-valued BSEs are likely to be related to the time since the last sample at a site. In theory, expected high-leverage sites shortly after sampling would become expected low-leverage sites; then, if they are not sampled through time, the expected leverage creeps upward again. This process repeats throughout the landscape, providing a dynamic map of expected BSE values into the future (S1 Text).

## A dynamic system of incentives

Our framework would be able to update on a monthly, weekly, daily, or even real-time basis, dependent on the taxa in question and the participation rates of the citizen science project. This dynamic aspect of citizen science projects is not novel. Many citizen science projects dynamically provide feedback to participants [27,63–65], often in the form of leaderboards, creating either friendly competition or a sense of self-competition by providing participants with performance feedback [27,66]. These tools may help sustain engagement over time [67], and similar approaches of incentivizing more sampling have proven successful with eBird participants [68].

**Fig 1:**
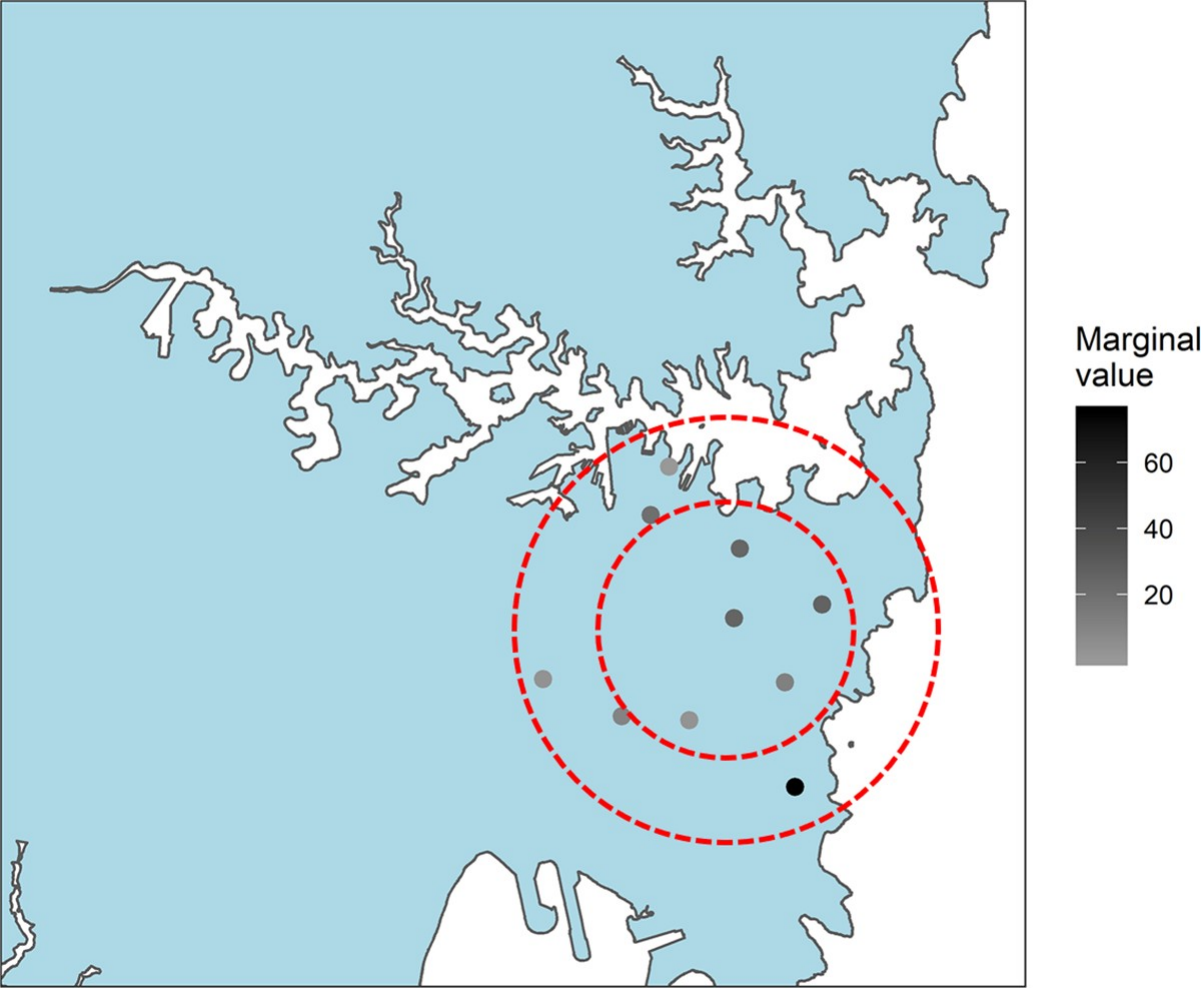
A potential map that users could be presented with, demonstrating the relative value of sites within their user-specified distance they are willing to sample (the dotted lines). The small circle could represent weekday sampling, whereas the larger circle could represent weekend sampling. Each site would be dynamically updated based on other participants’ submitted BSEs (S1 Text). Associated point values could be assigned relative to the priority level, and these point values could contribute to a “leaderboard” that prescribes scores based on the value of a given BSE. See here for a dynamic version showing the change in value through time. BSE, biodiversity sampling event.

In order to maximize observation value, we propose that leaderboards should not dwell on “total number of records” or “total number of species” but should also incorporate “overall value of participants’ observations.” One key concern about incentivizing science is to maintain data quality while directing effort more productively. To this end, we argue that incentives should be placed on sampling particular places at particular times, not actually finding specific species. To date, this is not common practice: other citizen science projects reward finding particular species (e.g., Questagame; https://questagame.com/). We argue that incentivizing looking rather than finding would ultimately decrease the ability of participants to “game the system” [69]. There is less likelihood of biases to exist resulting from individuals preferentially chasing rare species. We envision an approach that would incentivize the concept of submitting more “valuable” BSEs, encouraging participants to travel to sites that are prioritized based on the marginal value of a BSE from a specific site. Such an approach could see the following workflow (Fig 1):

Step 1: Citizen science participants could opt into the “challenge,” protecting privacy concerns. This ensures that only participants who are interested in participating would potentially get push notifications and enter potentially sensitive information about where they are willing to sample (see Step 2).
Step 2: Participants could provide a point (and an associated radius) on a map, indicating their preferred sampling area from which to submit BSEs. This could be flexible (e.g., intraweek variation), and when a participant is in a new area (i.e., on a vacation), they could provide updated areas.
Step 3: All potential sampling locations within the user-specified sampling domain could be selected, normalized, and ranked based on the citizen science project’s preferred weighting of the formula. This could then be presented as a map showing the highest-valued sites. An important first step would be to use spatial datasets to delineate the boundaries of private land tenure and sensitive areas, ensuring that participants are not encouraged to trespass on private property or disturb sensitive habitat. We also envision an approach in which users could opt in to receive push notifications that provide a ranked list of sites. This would be dynamically updated based on observations submitted on a given timescale.
Step 4: A given BSE could then be assigned “points,” and a leaderboard displaying the participant’s value of their submitted BSEs could be developed, encouraging further participation. Points could be assigned based on the prioritized site list, with the highest prioritized site receiving the most points, through to the least prioritized site (e.g., S1 Text). All BSEs submitted could be quantified, whereby any opportunistic BSE still receives a value, but just proportional to the potential value, should the participant decide to go sample at a site with the highest marginal value. The leaderboard would need to be normalized to the density of participants in a given area to keep participants’ scores on a comparable scale.

## Working within real-world constraints

Real-world constraints will inevitably limit the move toward optimal sampling of biodiversity in space and time. First, people are unevenly distributed across the globe, and wealth and literacy of the global population likely influence participation rates in citizen science projects. Second, groups of organisms (e.g., birds, fish, invertebrates) vary in their popularity with the general public both among [21] and within [22] taxa, influencing the level of participation in citizen science projects. Observations of some species (i.e., those that are less “popular” with the general public) may, therefore, be more valuable than others, but we do not include a species-specific resolution in our framework for assigning value to BSEs, because the species being detected cannot be predicted. Rather, the probability of a species being detected is a function of spatial and temporal sampling [70,71]. Third, citizen scientists are also more likely to sample in convenient locations. Indeed, one reason for the vast success of semistructured citizen science projects is the relative ease of data collection by the participants (i.e., few protocols to follow). Finally, not all citizen scientists contribute equally valuable contributions. This results from a difference in skills among observers—which can be accounted for during analyses [20]—and a difference in their dedication to a particular project. Moreover, motivations of participants vary among projects [72, 73]. For at least a subset of citizen scientists, a primary motivation of participation is to contribute to science [72, 73], suggesting that these participants are likely willing to improve their sampling, knowing it would benefit science. And there are potential education opportunities that can improve participants’ knowledge of how data are used by citizen science projects [5]. Other participants could be incentivized by providing “leaderboards” of participants with the most valuable BSEs. Although we cannot account for all of the above constraints, we can attempt to maximize the collective citizen science effort by optimizing when and where people sample biodiversity.

## Conclusions

Citizen science is mainstream, and research will increasingly use citizen science data at least in part to increase the spatial and temporal context of our research efforts. But are we maximizing the absolute power of the vast number of citizen scientists contributing to our collective knowledge? We think not. Although we provide intuitive and simplistic conceptualization, we acknowledge that this is only one way in which to “value” a given citizen scientist’s effort—which will ultimately be dependent on the project’s design and intended outcomes. Our goal in writing this piece is simple: we urge those developing and overseeing citizen science projects to think critically about refining data collection techniques, realizing the full potential for citizen science.

## Supporting information

S1 Text An example of a dynamic system to calculate marginal value. A guide to how we envision a dynamic system that can be used to calculate the value of BSEs in the future, broken down by steps, with figures and tables. This is intended as an example of how our framework could be implemented and is not intended to be prescriptive. We show it for a given, particular date, but this would be calculated on an updated, dynamic basis. BSE, biodiversity sampling event.
(PDF)

## References

[1] Soroye P, Ahmed N, Kerr JT. Opportunistic citizen science data transform understanding of species distributions, phenology, and diversity gradients for global change research. Global Change Biology. Global Change Biology. 2018;24(11): 5281–5291. 10.1111/gcb.14358 29920854

[2] Chandler M, See L, Copas K, Bonde AM, López BC, Danielsen F, et al Contribution of citizen science towards international biodiversity monitoring. Biological Conservation. 2017;213: 280–294.

[3] Dickinson JL, Shirk J, Bonter D, Bonney R, Crain RL, Martin J, et al The current state of citizen science as a tool for ecological research and public engagement. Frontiers in Ecology and the Environment. 2012;10: 291–297.

[4] McKinley DC, Miller-Rushing AJ, Ballard HL, Bonney R, Brown H, Cook-Patton SC, et al Citizen science can improve conservation science, natural resource management, and environmental protection. Biological Conservation. 2017;208: 15–28.

[5] Jordan RC, Gray SA, Howe DV, Brooks WR, Ehrenfeld JG. Knowledge gain and behavioral change in citizen-science programs. Conservation Biology. 2011;25: 1148–1154. 10.1111/j.1523-1739.2011.01745.x 21967292

[6] Starr J, Schweik CM, Bush N, Fletcher L, Finn J, Fish J, et al Lights, camera… citizen science: Assessing the effectiveness of smartphone-based video training in invasive plant identification. PLoS ONE. 2014;9: e111433 10.1371/journal.pone.0111433 25372597 PMC4221027

[7] Sullivan BL, Wood CL, Iliff MJ, Bonney RE, Fink D, Kelling S. EBird: A citizen-based bird observation network in the biological sciences. Biological Conservation. 2009;142: 2282–2292.

[8] Flemons P, Guralnick R, Krieger J, Ranipeta A, Neufeld D. A web-based gis tool for exploring the world’s biodiversity: The global biodiversity information facility mapping and analysis portal application (gbif-mapa). Ecological informatics. 2007;2: 49–60.

[9] Pocock MJ, Chandler M, Bonney R, Thornhill I, Albin A, August T, et al A vision for global biodiversity monitoring with citizen science. Advances in Ecological Research. 2018;59: 169–223.

[10] Kelling S, Johnston A, Bonn A, Fink D, Ruiz-Gutierrez V, Bonney R, et al Using semistructured surveys to improve citizen science data for monitoring biodiversity. BioScience. 2019;69: 170–179. 10.1093/biosci/biz010 30905970 PMC6422830

[11] Welvaert M, Caley P. Citizen surveillance for environmental monitoring: Combining the efforts of citizen science and crowdsourcing in a quantitative data framework. SpringerPlus. 2016;5: 1890 10.1186/s40064-016-3583-5 27843747 PMC5084151

[12] Van Horn G, Mac Aodha O, Song Y, Cui Y, Sun C, Shepard A, et al The inaturalist species classification and detection dataset In: Proceedings of the IEEE conference on computer vision and pattern recognition. Salt Lake City, Utah: IEEE; 2018 p. 8769–8778.

[13] Prudic KL, McFarland KP, Oliver JC, Hutchinson RA, Long EC, Kerr JT, et al EButterfly: Leveraging massive online citizen science for butterfly conservation. Insects. Multidisciplinary Digital Publishing Institute; 2017;8: 53. 10.3390/insects8020053 PMC5492067 28524117

[14] Rowley JJL, Callaghan CT, Cutajar T, Portway C, Potter K, Mahony S. FrodID: Citizen scientists provide validated biodiversity data on australia’s frogs. Herpetological Conservation and Biology. 2019;14: 155–170.

[15] Fox R, Warren MS, Brereton TM, Roy DB, Robinson A. A new red list of british butterflies. Insect Conservation and Diversity. 2011;4: 159–172.

[16] Boakes EH, McGowan PJ, Fuller RA, Chang-qing D, Clark NE, O’Connor K, et al Distorted views of biodiversity: Spatial and temporal bias in species occurrence data. PLoS Biol. 2010;8: e1000385 10.1371/journal.pbio.1000385 20532234 PMC2879389

[17] Beck J, Böller M, Erhardt A, Schwanghart W. Spatial bias in the gbif database and its effect on modeling species’ geographic distributions. Ecological Informatics. 2014;19: 10–15.

[18] Bird TJ, Bates AE, Lefcheck JS, Hill NA, Thomson RJ, Edgar GJ, et al Statistical solutions for error and bias in global citizen science datasets. Biological Conservation. 2014;173: 144–154.

[19] Courter JR, Johnson RJ, Stuyck CM, Lang BA, Kaiser EW. Weekend bias in citizen science data reporting: Implications for phenology studies. International journal of biometeorology. 2013;57: 715–720. 10.1007/s00484-012-0598-7 23104424

[20] Kelling S, Johnston A, Hochachka WM, Iliff M, Fink D, Gerbracht J, et al Can observation skills of citizen scientists be estimated using species accumulation curves? PLoS ONE. 2015;10: e0139600 10.1371/journal.pone.0139600 26451728 PMC4599805

[21] Mair L, Ruete A. Explaining spatial variation in the recording effort of citizen science data across multiple taxa. PLoS ONE. 2016;11: e0147796 10.1371/journal.pone.0147796 26820846 PMC4731209

[22] Ward DF. Understanding sampling and taxonomic biases recorded by citizen scientists. Journal of insect conservation. 2014;18: 753–756.

[23] Gilfedder M, Robinson CJ, Watson JE, Campbell TG, Sullivan BL, Possingham HP. Brokering trust in citizen science. Society & Natural Resources. 2019;32: 292–302.

[24] Johnston A, Fink D, Hochachka WM, Kelling S. Estimates of observer expertise improve species distributions from citizen science data. Methods in Ecology and Evolution. 2018;9: 88–97.

[25] Isaac NJ, Strien AJ van, August TA, Zeeuw MP de, Roy DB. Statistics for citizen science: Extracting signals of change from noisy ecological data. Methods in Ecology and Evolution. 2014;5: 1052–1060.

[26] Robinson OJ, Ruiz-Gutierrez V, Fink D. Correcting for bias in distribution modelling for rare species using citizen science data. Diversity and Distributions. 2018;24: 460–472.

[27] Wiggins A, Crowston K. From conservation to crowdsourcing: A typology of citizen science In: 2011 44th Hawaii international conference on system sciences. 2011; Kauai, Hawaii. Piscataway, NJ: IEEE; 2011 p. 1–10.

[28] Wiggins A, Newman G, Stevenson RD, Crowston K. Mechanisms for data quality and validation in citizen science In: Proceedings of the 2011 ieee seventh international conference on e-science workshops. Piscataway, NJ: IEEE; 2011 p. 14–19.

[29] Fithian W, Elith J, Hastie T, Keith DA. Bias correction in species distribution models: Pooling survey and collection data for multiple species. Methods in Ecology and Evolution. 2015;6: 424–438. 10.1111/2041-210X.12242 27840673 PMC5102514

[30] Giraud C, Calenge C, Coron C, Julliard R. Capitalizing on opportunistic data for monitoring relative abundances of species. Biometrics. 2016;72: 649–658. 10.1111/biom.12431 26496390

[31] Fink D, Hochachka WM. Using data mining to discover biological patterns in citizen science observations In: Dickinson JL, Bonney R, editors. Citizen science: public participation in environmental research. Ithaca, NY: Comstock Publishing Associates; 2012 p. 125–138.

[32] Hochachka WM, Fink D, Hutchinson RA, Sheldon D, Wong W-K, Kelling S. Data-intensive science applied to broad-scale citizen science. Trends in ecology & evolution. 2012;27: 130–137. 22192976 10.1016/j.tree.2011.11.006

[33] Kelling S, Fink D, La Sorte FA, Johnston A, Bruns NE, Hochachka WM. Taking a ‘big data’ approach to data quality in a citizen science project. Ambio. 2015;44: 601–611. 10.1007/s13280-015-0710-4 PMC4623867 26508347

[34] Guillera-Arroita G, Lahoz-Monfort JJ, Elith J, Gordon A, Kujala H, Lentini PE, et al Is my species distribution model fit for purpose? Matching data and models to applications. Global Ecology and Biogeography. 2015;24: 276–292.

[35] Fink D, Damoulas T, Bruns NE, La Sorte FA, Hochachka WM, Gomes CP, et al Crowdsourcing meets ecology: Hemisphere-wide spatiotemporal species distribution models. AI magazine. 2014;35: 19–30.

[36] Fink D, Hochachka WM, Zuckerberg B, Winkler DW, Shaby B, Munson MA, et al Spatiotemporal exploratory models for broad-scale survey data. Ecological Applications. 2010;20: 2131–2147. 21265447 10.1890/09-1340.1

[37] Jones BL, Unsworth RK, McKenzie LJ, Yoshida RL, Cullen-Unsworth LC. Crowdsourcing conservation: The role of citizen science in securing a future for seagrass. Marine pollution bulletin. 2018;134: 210–215. 10.1016/j.marpolbul.2017.11.005 29137812

[38] Silvertown J, Harvey M, Greenwood R, Dodd M, Rosewell J, Rebelo T, et al Crowdsourcing the identification of organisms: A case-study of iSpot. ZooKeys. 2015; 125–146. 10.3897/zookeys.480.8803 PMC4319112 25685027

[39] Zilli D, Parson O, Merrett GV, Rogers A. A hidden markov model-based acoustic cicada detector for crowdsourced smartphone biodiversity monitoring. Journal of Artificial Intelligence Research. 2014;51: 805–827.

[40] See L, Mooney P, Foody G, Bastin L, Comber A, Estima J, et al Crowdsourcing, citizen science or volunteered geographic information? The current state of crowdsourced geographic information. ISPRS International Journal of Geo-Information. 2016;5: 55.

[41] Tulloch AI, Possingham HP, Joseph LN, Szabo J, Martin TG. Realising the full potential of citizen science monitoring programs. Biological Conservation. 2013;165: 128–138.

[42] Yoccoz NG, Nichols JD, Boulinier T. Monitoring of biological diversity in space and time. Trends in Ecology & Evolution. 2001;16: 446–453.

[43] Bradsworth N, White JG, Isaac B, Cooke R. Species distribution models derived from citizen science data predict the fine scale movements of owls in an urbanizing landscape. Biological conservation. 2017;213: 27–35.

[44] Strien AJ van, Swaay CA van, Termaat T. Opportunistic citizen science data of animal species produce reliable estimates of distribution trends if analysed with occupancy models. Journal of Applied Ecology. 2013;50: 1450–1458.

[45] Tiago P, Pereira HM, Capinha C. Using citizen science data to estimate climatic niches and species distributions. Basic and Applied Ecology. 2017;20: 75–85.

[46] Stuart-Smith RD, Edgar GJ, Barrett NS, Bates AE, Baker SC, Bax NJ, et al Assessing national biodiversity trends for rocky and coral reefs through the integration of citizen science and scientific monitoring programs. Bioscience. 2017;67: 134–146. 10.1093/biosci/biw180 28596615 PMC5384302

[47] Bahls LL. New diatoms from the american west—A tribute to citizen science. Proceedings of the Academy of Natural Sciences of Philadelphia. BioOne; 2014;163: 61–85.

[48] Solla SR de, Shirose LJ, Fernie KJ Barrett GC, Brousseau CS Bishop CA. Effect of sampling effort and species detectability on volunteer based anuran monitoring programs. Biological Conservation. 2005;121: 585–594.

[49] La Sorte FA, Tingley MW, Hurlbert AH. The role of urban and agricultural areas during avian migration: An assessment of within-year temporal turnover. Global ecology and biogeography. 2014;23: 1225–1234.

[50] Supp S, La Sorte FA, Cormier TA, Lim MC, Powers DR, Wethington SM, et al Citizen-science data provides new insight into annual and seasonal variation in migration patterns. Ecosphere. 2015;6: 1–19.

[51] Pocock MJ, Roy HE, Fox R, Ellis WN, Botham M. Citizen science and invasive alien species: Predicting the detection of the oak processionary moth thaumetopoea processionea by moth recorders. Biological conservation. 2017;208: 146–154.

[52] Grason EW, McDonald PS, Adams J, Litle K, Apple JK, Pleus A. Citizen science program detects range expansion of the globally invasive european green crab in washington state (usa). Management of Biological Invasions. 2018;9: 39–47.

[53] Horns JJ, Adler FR, Şekercioğlu ÇH. Using opportunistic citizen science data to estimate avian population trends. Biological conservation. 2018;221: 151–159.

[54] Dennis EB, Morgan BJ, Brereton TM, Roy DB, Fox R. Using citizen science butterfly counts to predict species population trends. Conservation Biology. 2017;31: 1350–1361. 10.1111/cobi.12956 28474803

[55] MacArthur RH, Wilson EO. The theory of island biogeography Princeton, NJ: Princeton University Press; 1967.

[56] Connor EF, McCoy ED. The statistics and biology of the species-area relationship. The American Naturalist. 1979;113: 791–833.

[57] Crawley M, Harral J. Scale dependence in plant biodiversity. Science. American Association for the Advancement of Science; 2001;291: 864–868. 10.1126/science.291.5505.864 11157164

[58] MacKenzie DI, Kendall WL. How should detection probability be incorporated into estimates of relative abundance? Ecology. 2002;83: 2387–2393.

[59] Nichols JD, Hines JE, Sauer JR, Fallon FW, Fallon JE, Heglund PJ. A double-observer approach for estimating detection probability and abundance from point counts. The Auk. 2000;117: 393–408.

[60] Callaghan C, Lyons M, Martin J, Major R, Kingsford R. Assessing the reliability of avian biodiversity measures of urban greenspaces using eBird citizen science data. Avian Conservation and Ecology. 2017;12.

[61] Cooper CB, Dickinson J, Phillips T, Bonney R. Citizen science as a tool for conservation in residential ecosystems. Ecology and Society. 2007;12.

[62] Cook RD. Detection of influential observation in linear regression. Technometrics. 1977;19: 15–18.

[63] Bowser A, Hansen D, He Y, Boston C, Reid M, Gunnell L, et al Using gamification to inspire new citizen science volunteers In: Proceedings of the first international conference on gameful design, research, and applications. 2013; Stratford, Canada. New York, NY: ACM; 2013 p. 18–25.

[64] Ponti M, Hillman T, Kullenberg C, Kasperowski D. Getting it right or being top rank: Games in citizen science. Citizen Science: Theory and Practice. 2018;3(1):1–12.

[65] Wood C, Sullivan B, Iliff M, Fink D, Kelling S. EBird: Engaging birders in science and conservation. PLoS Biol. 2011;9: e1001220 10.1371/journal.pbio.1001220 22205876 PMC3243722

[66] Wiggins A, Crowston K. Surveying the citizen science landscape. First Monday. 2015;20.

[67] Iacovides I, Jennett C, Cornish-Trestrail C, Cox AL. Do games attract or sustain engagement in citizen science?: A study of volunteer motivations In: CHI’13 extended abstracts on human factors in computing systems. 2013; Paris, France. New York, NY: ACM; 2013 p. 1101–1106.

[68] Xue Y, Davies I, Fink D, Wood C, Gomes CP. Avicaching: A two stage game for bias reduction in citizen science In: Proceedings of the 2016 international conference on autonomous agents & multiagent systems. Richland, SC: International Foundation for Autonomous Agents and Multiagent Systems; 2016 p. 776–785.

[69] O’Grady MJ, Muldoon C, Carr D, Wan J, Kroon B, O’Hare GM. Intelligent sensing for citizen science. Mobile Networks and Applications. 2016;21: 375–385.

[70] Colwell RK, Mao CX, Chang J. Interpolating, extrapolating, and comparing incidence-based species accumulation curves. Ecology. 2004;85: 2717–2727.

[71] Pärtel M, Szava-Kovats R, Zobel M. Dark diversity: Shedding light on absent species. Trends in ecology & evolution. 2011;26: 124–128. 21195505 10.1016/j.tree.2010.12.004

[72] Rotman D, Preece J, Hammock J, Procita K, Hansen D, Parr C, et al Dynamic changes in motivation in collaborative citizen-science projects In: Proceedings of the ACM 2012 conference on computer supported cooperative work. 2012; Seattle, Washington. New York, NY: ACM; 2012 p. 217–226.

[73] Tiago P, Gouveia MJ, Capinha C, Santos-Reis M, Pereira HM. The influence of motivational factors on the frequency of participation in citizen science activities. Nature Conservation. 2017;18: 61–78.

