# Supplementary material for: Improving big citizen science data: Moving beyond haphazard sampling
Source: PLoS Biol. 2019 Jun 27;17(6):e3000357. doi: 10.1371/journal.pbio.3000357 (PMC6619805; doi:10.1371/journal.pbio.3000357)
Supplement: S1 Text — A guide to how we envision a dynamic system that can be used to calculate the value of BSEs in the future, broken down by steps, with figures and tables. This is intended as an example of how our framework could be implemented and is not intended to be prescriptive. We show it for a given, particular date, but this would be calculated on an updated, dynamic basis. BSE, biodiversity sampling event. (PDF) [file pbio.3000357.s001.pdf]

Step 1. Choose sites that are within a user-specified region, defined by use-specified point and associated radius. For our example, we chose ten urban greenspaces in the Sydney region. This is intended as an example of how our framework could be implemented, and not intended to be prescriptive. We show it for a given, particular date, but this would be calculated on an updated, dynamic basis. [This GitHub repository]() hosts the data and code necessary to produce the below outputs.

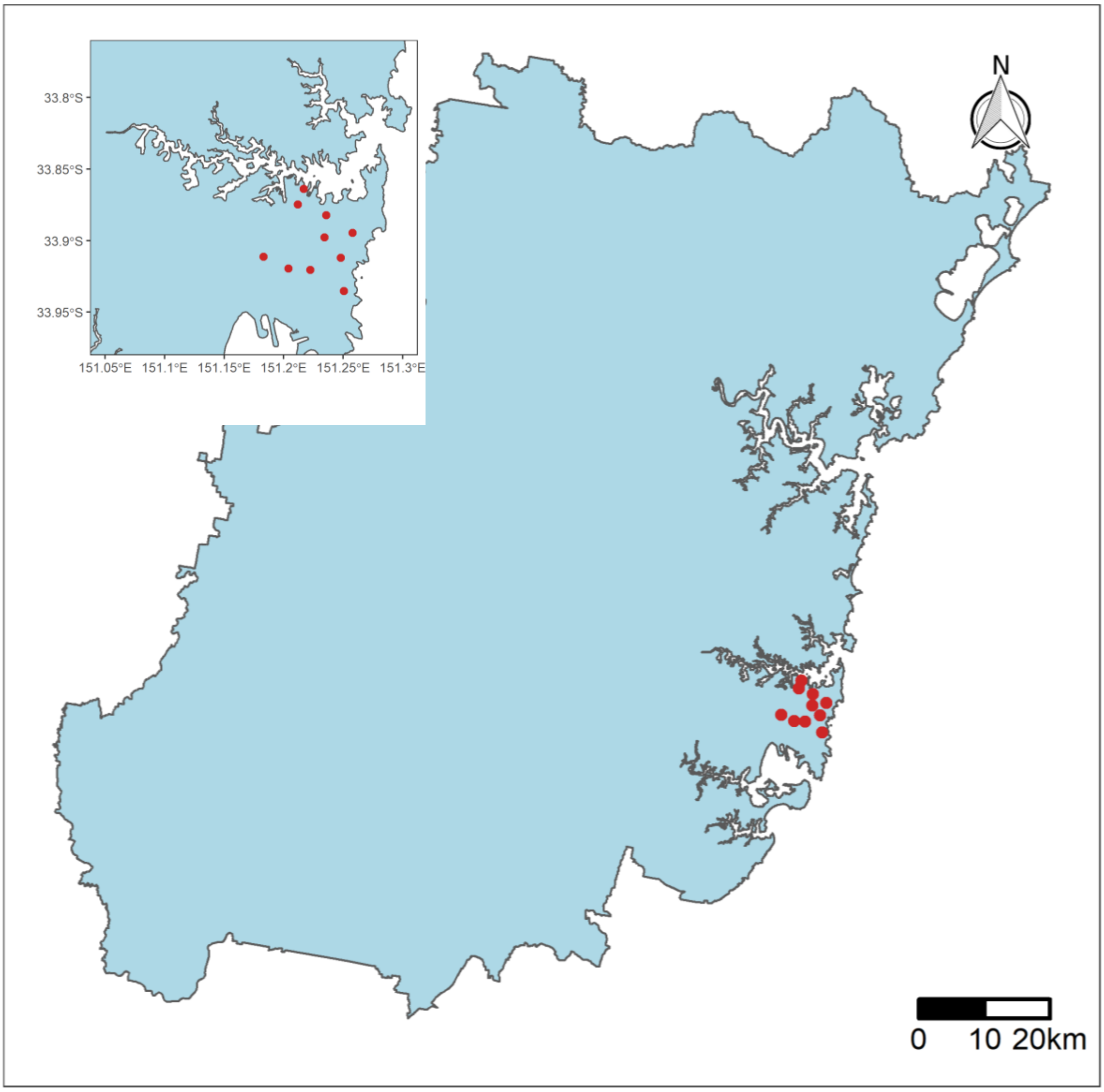

Step 2. Define the potential parameters to calculate for each of the sites. For our example, we chose: (1) whether a site was sampled or not; (2) distance from the nearest sampled site (in km); (3) median sampling interval between BSEs at a site (measured in number of days); (4) days between first and last observation, i.e., the total duration of sampling at a site; (5) median sampling interval of the nearest neighbour (measured in days); and (6) days since last sampling event at a site. We used eBird data between January 1, 2010 and December 31st, 2018. See this GitHub repository for code and data to reproduce these results. We ran this code for a specific date (November 22nd, 2018), and the results are in the table below.

LOCALITY_ID	Sampled	NEIGHBOR_ID	Dist_km	Median_sampling_interval	Duration_samples	Days_since_last_sample	Neighbor_sampling_interval
L1030678	yes	L1300136	2.358412	588	1176	494	10
L915566	yes	L1300136	1.279883	4	3139	0	10
L945869	yes	L8083126	2.212982	1	3156	0	NA
L2444301	yes	L2557723	5.99674	8	2361	9	85
L1300136	yes	L1030678	2.358412	10	2963	7	588
L3007885	no	L5146094	3.095377	NA	NA	NA	5
L2557723	yes	L2444301	5.99674	85	1279	285	8
L5076722	yes	L5146094	1.67542	36	481	258	5
L5146094	yes	L5076722	1.67542	5	669	39	36
L8083126	yes	L945869	2.212982	NA	NA	25	1

Step 3. Note that there are "NAs" in the summary. This results from a site having never been sampled, and thus it will not have a median sampling interval. Further, if a site has only been sampled 1 or 2 times, it will not have a median sampling interval or a duration of sampling (e.g., L8083126). Therefore, we need to define the NAs. To do this, we assign the NAs to 1 standard deviation above the mean for that given column. This can be done in different ways, depending on the relative importance of the parameter to a citizen science project. This provides an updated summary which looks like:

LOCALITY_ID	Sampled	NEIGHBOR_ID	Dist_km	Median_sampling_interval	Duration_samples	Days_since_last_sample	Neighbor_sampling_interval
L1030678	yes	L1300136	2.358412	588	1176	494	10
L915566	yes	L1300136	1.279883	4	3139	0	10
L945869	yes	L8083126	2.212982	1	3156	0	970.3695
L2444301	yes	L2557723	5.99674	8	2361	9	85
L1300136	yes	L1030678	2.358412	10	2963	7	588
L3007885	no	L5146094	3.095377	992.6691	5410.369	851.3996	5
L2557723	yes	L2444301	5.99674	85	1279	285	8
L5076722	yes	L5146094	1.67542	36	481	258	5
L5146094	yes	L5076722	1.67542	5	669	39	36
L8083126	yes	L945869	2.212982	992.6691	5410.369	25	1

Step 4. If a site was sampled, then that site received a value of 0, and if it was unsampled it received a value of 1. This means that if all sites were sampled in a suite of potential sites, then that parameter would 'drop-out' of the process. This value was multiplied by the distance to the nearest sampled site, meaning that if more than one unsampled site was possible, the site with the greatest distance to the nearest sampled site would have higher 'value'. We also multiplied the median sampling interval by the duration of sampling days at a site. This provides a weighting measure whereby sites which have been sampled for a long time receive more potential value than sites sampled for a short period of time. The other parameters remained the same without any additional changes.

Because each potential sampling site is a possible choice in a dynamic framework, each site can be considered relative to one another, on any given potential sampling day. This means, that for simplicity, the parameters can be normalized to a particular range. In this example we scale each of the four parameters (collapsed from 6) to a range of 0-25. This is an important step, because if users wanted to place more 'value' on a given parameter than they could manually manipulate these parameters. These parameters can also be quantitatively assigned by using the leverage approach from a statistical model, highlighting which parameters are most significant for a given desired outcome of the citizen science data (i.e., a given statistical model such as species distribution model or trend detection model). In our example, we weighted each equally, for simplicity. The normalized variables to a specific scale look like:

LOCALITY_ID	norm.distance_sample	norm.m_s_i	norm.m_s_i_n_n	norm.days_since
L1030678	0.00	3.21	0.23	14.51
L915566	0.00	0.04	0.23	0.00
L945869	0.00	0.00	25.00	0.00
L2444301	0.00	0.07	2.17	0.26
L1300136	0.00	0.12	15.14	0.21
L3007885	25.00	25.00	0.10	25.00
L2557723	0.00	0.49	0.18	8.37
L5076722	0.00	0.07	0.10	7.58
L5146094	0.00	0.00	0.90	1.15
L8083126	0.00	25.00	0.00	0.73

Only one site was not sampled (L3007885), and thus it received a value of "25", the highest in this parameter. The rest of the variables are now normalized on the same scale, relative to one another.

Step 5. One can simply 'add' the columns together to receive an overall value of a given site, based on the parameters chosen. Importantly, by scaling the parameters differently, citizen science project managers can decide which parameters are most important for their intended outcomes. Ultimately, this should be driven by the leverage in a statistical modelling framework. Here, we can show the values of the sites for November 22nd, 2018, based on our pre-defined parameters. The unsampled site has the highest value in our current framework demonstration.

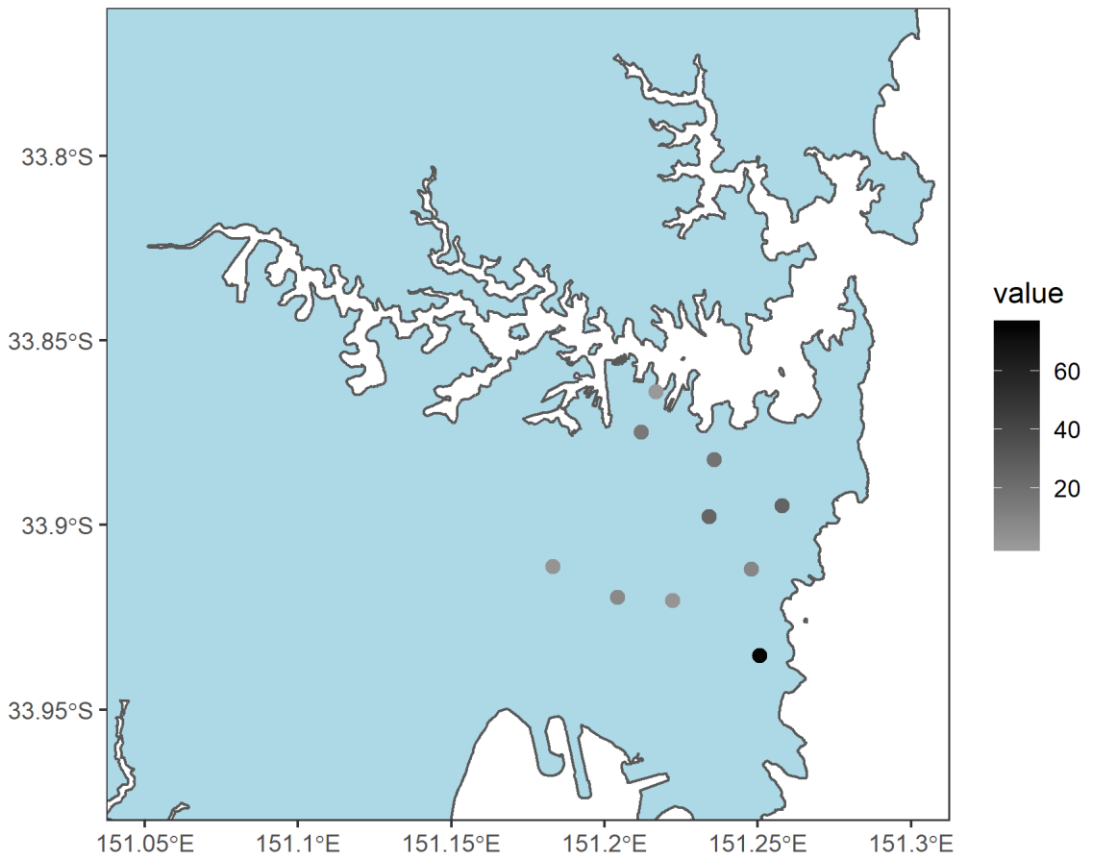

Step 6. This is done dynamically, through time, as more BSEs are submitted to a citizen science project. To illustrate this, we made a gif of these values changing through time. Although the values do not change to a large extent in our example, one can visualize these changes [here](#).
